# High-level fed-batch fermentative expression of an engineered *Staphylococcal* protein A based ligand in *E. coli*: purification and characterization

**DOI:** 10.1186/s13568-015-0155-y

**Published:** 2015-11-10

**Authors:** Martin Kangwa, Vikas Yelemane, Ayse Nur Polat, Kanaka Durga Devi Gorrepati, Mariano Grasselli, Marcelo Fernández-Lahore

**Affiliations:** Downstream Bioprocessing Laboratory, Department of Life Sciences and Chemistry, Jacobs University, Campus Ring 1, 28759 Bremen, Germany; Laboratorio de Materiales Biotecnológicos (LaMaBio), Universidad Nacional de Quilmes-IMBICE (CONICET), Roque Sáenz Peña 352, B1876BXD Bernal, Argentina

**Keywords:** High cell density, Fed-batch fermentation, Recombinant protein A, *E. coli*, *AviPure*

## Abstract

The major platform for high level recombinant protein production is based on genetically modified microorganisms like *Escherichia coli* (*E. coli*) due to its short dividing time, ability to use inexpensive substrates and additionally, its genetics is comparatively simple, well characterized and can be manipulated easily. Here, we investigated the possibilities of finding the best media for high cell density fermentation, by analyzing different media samples, focusing on improving fermentation techniques and recombinant protein production. Initial fermentation of *E. coli* BL21 DE3:pAV01 in baffled flasks showed that high cell density was achieved when using complex media, Luria–Bertani (LB) and Terrific medium broth (TB) (10 and 14 g/L wet weight, respectively), as compared to mineral media M9, modified minimal medium (MMM) and Riesenberg mineral medium (RM) (7, 8 and 7 g/L, respectively). However, in fed-batch fermentation processes when using MMM after 25 h cultivation, it was possible to yield an optical density (OD_600_) of 139 corresponding to 172 g/L of wet biomass was produced in a 30 L TV Techfors-S Infors HT fermenter, with a computer controlled nutrient supply (glucose as a carbon source) delivery system, indicating nearly 1.5 times that obtained from TB. Upon purification, a total of 1.65 mg/g of protein per gram cell biomass was obtained and the purified AviPure showed affinity for immunoglobulin. High cell density fed batch fermentation was achieved by selecting the best media and growth conditions, by utilizing a number of fermentation parameters like media, fermentation conditions, chemical concentrations, pO_2_ level, stirrer speed, pH level and feed media addition. It is possible to reach cell densities higher than shake flasks and stirred tank reactors with the improved oxygen transfer rate and feed.

**Electronic supplementary material:**

The online version of this article (doi:10.1186/s13568-015-0155-y) contains supplementary material, which is available to authorized users.

## Introduction

The current high demand, coupled with therapeutic potential of biopharmaceuticals, go hand in hand with the demand in improving industrial biotechnological processes to produce biopharmaceutical molecules of higher quality and quantity. The production of biopharmaceuticals like therapeutic proteins via fermentation processes is not critical as intensive efforts have been put into their production. For instance, antibody titers in mammalian cell culture has increased drastically over the last decades and currently, it is possible to obtain titers more than 8 g/L, and protein expression levels higher than 15 g/L (Li et al. 2010). However, the mass throughput in biotechnological production plant is limited due to the necessary requirements in the purification of products, in which the costs of production of monoclonal antibodies are mostly defined by the costs of the downstream processing. The most dominating costs is chromatography, filter materials, chemicals and water (Chen et al. 1997; Chin et al. 2015; Glazyrina et al. 2010; Hao et al. 2013). Thus, innovative solutions and technologies in regard to production times, costs and resources have to be developed.

Monoclonal antibodies are attractive tools to develop therapeutics due to their multiple applications. They are among the most promising compounds on the market and one of the pipelines of pharmaceutical biotechnology (Carter 2011; Kabir 2002). They can be employed in vivo according to their ability to bind specifically to a target molecule. In monoclonal antibody purification, affinity chromatography has commonly been used with *Staphylococcalaureus* protein A (SpA) as the ligand. Protein A is a type I membrane protein from the bacteria *Staphylococcus aureus* (Bratkovic et al. 2006; Freiherr von Roman et al. 2014; Jungbauer and Hahn 2004; Tsukamoto et al. 2014), consisting of five domains that have a high affinity for the fragment crystallizable (Fc) region of antibodies (Moks et al. 1986; Pabst et al. 2014; Romagnani et al. 1982). The SpA molecule consists of a single polypeptide chain that folds into helix bundles with a molecular weight of approximately 42 kDa. Due to high selectivity and good physiochemical stability protein A is a preferred generic ligand for affinity purification of antibodies and molecules tagged with an antibody Fc region. For this reason the molecule have been used for several immunological, and purification applications (Asenjo and Andrews 2009; Barroso et al. 2014; Boi et al. 2009; Zamolo et al. 2008; Zhang et al. 2015), therefore there is need for high level production of the protein.

The currently used protein production technology is based on genetically modified microorganisms such as *Escherichia coli* (*E. coli*) (Shiloach and Fass 2005; Zhang et al. 2004). It is one of the most widely used host cells in recombinant protein production and metabolic engineering, due to its short dividing time, ability to use inexpensive substrates and additionally, the genetics of *E. coli* are comparatively simple, well characterized and can easily be manipulated (Glazyrina et al. 2010; Yee and Blanch 1992).

The achievement of high cell concentrations and the use of recombinant *E. coli,* it is necessary to develop fed-batch strategies and multistage reactor systems for fermentation processes that provide high cell mass productivity and high stability. The high cell concentrations fermentation possess some advantages like, reduced reactor volumes, higher volumetric productivities, less efforts in up and downstream processing, reduced waste water and lower costs of production. Microbial fermentation can be categorized into three major groups: batch, fed-batch, and continuous (Chen et al. 1997; Glazyrina et al. 2012, 2010). Batch processes are suitable for small productions and the equipment is relatively simple compared to other processes. However, reaction conditions changes with time causing problems in precise fermentation processes, but on the other hand provide high production and a better quality product for continuous processes due to constant conditions (Shpigel et al. 2000; Yee and Blanch 1992; Cerrone et al. 2014; Ibrahim and Steinbuchel 2010). Batch processes are more useful for kinetic studies, and require flow control in order to maintain constant conditions (Shiloach and Fass 2005). The disadvantage of this method is that the cultures can be unstable after longer fermentation periods. Fed-batch processes primarily focus on increasing the biomass concentration and thereby increasing the productivity, while minimizing problems encountered in high cell density cultivations, since during microbial growth, nutrients, gasses, and trace elements (if necessary) are added (Freiherr von Roman et al. 2014; Glazyrina et al. 2012, 2010; Hoffmann et al. 2000; Korz et al. 1995; Krause et al. 2010). The volumetric yield of the recombinant product depends on both biomass concentrations and the specific cellular product yield.

In this study we focused on high-level fed-batch fermentative expression of an engineered SpA B domain based ligand in *E. coli* BL21-DE3 (Novagen, Madison, USA), followed by purification and characterization. Though SpA has five domains with affinity for the Fc region, the molecule shows incapability to simultaneously bind five antibody molecules due to steric hindrance that might be caused by bound antibodies which blocks the access of others to the binding sites. This problem of steric hindrance encountered with SpA can be solved by using an AviPure, a ligand analogue based on the native SpA B domain, with a lower molecular weight of approximately 14 kDa, containing two repeats of the SpA B domain, a histidine tag at the *N*-terminal for Ni–NTA based purification and two binding sites (Cysteine–Histidine) at the C-terminal for the immobilization via epoxy groups to the solid support.

## Materials and methods

### Protein engineering

AviPure with two domain repeats was designed based on *Staphylococcus aureus* M0464 SpA B domain (accession number: KT377029) with some modification at the *N*, linker and C-terminal and the codon optimized sequence for expression in *E. coli* BL21 (DE3) was synthesized by Eurofingenomics (Erlangen, Germany) (Additional file 1: Figure S1). Molecular sub-cloning of AviPure into pET28a (+) was performed. Oligonucleotide primers, Avi-Fw ACT AGC TAG CGG ATC CCT GGC GGA TAA TAA ATT TAA C containing *Bam*HI restriction site and Avi-Rv GAA TTC TGA AGC TTC CTT AGT GGC AAT GGC AAT G containing *Hin*dIII restriction site (underlined bases indicate *Bam*HI and *Hin*dIII sites, respectively) also synthesized by Eurofingenomics (Erlangen, Germany) were used to extract the *AviPure* gene, while restriction enzymes *Bam*HI-HF and *Hin*dIII-HF were purchased from New England Biolab (NEB, USA). The expression vector pET28a (+) was from Novagen (San Diego, CA, USA). AviPure PCR amplification was done using the Phusion^®^ High-Fidelity DNA Polymerase (NEB, USA) consisted of 36 cycles of 94 °C for 30 s, 60 °C for 40 s and 72 °C for 2 min, and followed by a final extension step at 72 °C for 5 min, producing PCR gene product of about 407 bp. Plasmid pET28a (+) and PCR products were purified using NucleoSpin^®^ Plasmid extraction and NucleoSpin^®^ Gel and PCR Clean-up kit (Macherey–Nagel GmbH and Co. KG, Düren, Germany). The pET28a (+) and amplified *AviPure* gene product were individually digested (*Bam*HI-HF and *Hin*dIII-HF) and ligated to obtain pAV01 with the *N*-terminal hexa-histidine tag that facilitate easy purification via Ni–NTA resin. The obtained expression vector was initially amplified in *E. coli* TOP10 cells and the correctness of the sequence was verified by analytical restriction digest and DNA sequencing (Eurofins MWG Operon, Ebersberg, Germany). The plasmid pAV01 was later isolated and subsequently transformed into *E. coli* BL21(DE3) cells to obtain the *E. coli* BL 21 (DE3):pAV01 for target protein expression.

### Nucleotide sequence accession numbers

Gene sequences generated in this study were deposited in GenBank and the accession numbers are KT377029.

### Protein expression

Five different media was analyzed for AviPure production: (1) Luria–Bertani medium broth (LB) (10 g/L bacto-tryptone, 5 g/L yeast extract, 5 g/L NaCl); (2) Terrific medium broth (TB) (12 g/L tryptone, 24 g/L yeast extract, 9.4 g/L K_2_HPO_4_, 2.2 g/L KH_2_PO_4_, 4 mL 98 % w/v glycerol); (3) M9 minimal medium (M9) (12.8 g/L Na_2_HPO_2_, 3 g/L KH_2_PO_4_, 0.5 g/L NaCl, 2 g/L NH_4_Cl, 20 g/L glucose, 0.01 g/L CaCl_2_, 0.12 g/L MgSO_4_, 0.002 g/L FeCl_3_); (4) Modified Minimal Medium (MMM) [30 g/L glucose, 1.2 g/L MgSO4 × 7H_2_O, 13.3 g/L KH_2_PO_4_, 4 g/L (NH_4_)_2_HPO_4_, 1.7 g/L citric acid, 10 mL of trace metals solution, 100 µL thiamine HCL (45 mg/mL)]; (5) Riesenberg Mineral Medium (RMM) (7.8 g/L KH_2_PO_4_, 2.33 g/L (NH_4_)_2_PO_4_, 10 g/L glucose, 0.5 g/L citric acid, 1.1 g/L MgSO_4_ × H_2_O, trace element solution, thiamine HCl.

*E. coli* BL 21 (DE3):pAV01 cells from a glycerol stock were initially plated on LB-agar plate containing appropriate antibiotics and incubated overnight at 37 °C, one single colony was later inoculated into 2 mL LB medium supplemented with appropriate antibiotics and cultured overnight at 37 °C with shaking at 225 rpm. The overnight culture then centrifuge and re-suspended in appropriate media, was transferred into Erlenmeyer flask containing 1 L cultures of each media and were incubated at 37 °C, at 225 rpm. All media were supplemented with 25 mg/L final amount of Kanamycin to avoid contaminations. Protein expression was induced by the addition of 1 mM final concentration Isopropyl β-D-1-thiogalactopyranoside (IPTG) at OD_600_ 0.5. Samples were taken before and after induction and both the supernatant and the pellets were analyzed in a 15 % SDS-PAGE.

### Mini-pilot scale fermentation of AviPure

For mini-pilot scale fermentation, TB and MMM were used. Initially, overnight cultures supplemented with appropriate antibiotics was prepared in 4 mL LB, further transferred and cultured to an OD_600_ of 1.0 in 500 mL Erlenmeyer flask of either TB or MMM media. The culture was later used to inoculate 9.5 L of TB and MMM media in a 30 L TV Techfors-S (Infors HT, Switzerland) fermenter previously sterilized at 121 °C for 20 min and cooled to 37 °C, respectively. During fermentation process some parameters were observed due to their importance; the change in OD, pH, aeration, antifoam, carbon source, agitation. At first the fermentation was run in batch mode with dissolved oxygen level maintained at required saturation (pO_2_) by using filtered air and with stirring speed in cascade mode in order to achieve and keep pO_2_ level constant. The production of foam was hindered by using 5 % antifoam solution (Sigma Aldrich, Germany) and the cultivation temperature was set at 37 °C. During fermentation, the pH of the media was maintained at 6.8 using a standard pH electrode (Mettler Toledo, USA) by the addition of phosphoric acid and liquid ammonia and monitored using the pH sensor unit. Before switching to fed-batch, the dissolved oxygen was used as a guide of the amount of feed needed (Yee and Blanch 1992), with the DO-stat used to balance the amount of glycerol/glucose needed at a time. Calibrated peristaltic pumps were used to control the feed rate for feed media (85 % glucose for MMM) which was determined by the metabolic rate of the culture. During fermentation, sampling of the culture in the medium was performed and analyzed for wet cell weight, and optical density at 600 nm. After 22 h, feed was reduced and protein expression was induced with 1 mM final concentration of IPTG solution. Cultivation was continued further 3 h till the end of cultivation time. *E.coli* cells pellets were collected by centrifugation using the Thermo Scientific Contifuge Stratos tabletop centrifuge, having a continuous flow rotor at 16,000 rpm, at 4 °C.

### Protein purification

For AviPure purification, 5 g biomass (pellets) from the previously mini-pilot scale fermentation was re-suspended in 40 mL sonication/Wash buffer (50 mM Phosphate Buffer pH 7.5, 300 mM NaCl, 10 mM Imidazole) and 350 µL protease inhibitor cocktail mix. Direct sonication was performed for 45 min using amplitude of 90 % and 0.6 cycles; with the beaker containing cell suspension placed in ice/ice cold water and was continuously stirred to keep cool. After sonication, the lysate was centrifuged for 1 h at 16,000 rpm using a Beckman Coulot Avanti J-E centrifuge to get rid of cell debris. IMAC was used to purify the target protein and a 5 mL Ni–NTA column volume was chosen, equilibrated with sonication/wash buffer before loading. After centrifugation, the final 40 mL supernatant was loaded on the column. UPC–900 Amersham Biosciences AKTA FPLC system was employed for the purification. The elution buffer used was the same as the sonication/wash buffer, but contained a higher imidazole concentration (250 mM). The obtained protein was analyzed by SDS-PAGE following the method of Laemmli.

### IgG affinity detection of AviPure via Western blot analysis

The affinity of the AviPure for IgG was investigated using protein affinity binding. Samples collected during cultivation (before and after induction) were resolved by a 15 % SDS-PAGE gel. Separated proteins were blotted onto nitrocellulose membranes (Whatman, Dassel, Germany) using a wet electro-blotting apparatus (Bio-Rad, USA). To prevent non-specific binding, the free binding sites of the membranes were saturated with 5 % fat free dry milk powder in TTBS (overnight at 4 °C with gentle agitation). Thereafter, the membrane was thoroughly washed five times, each for 5 min with 50 mL TTBS. After each incubation, the membrane was washed in the same way. The Goat anti-protein A polyclonal antibodies HRP conjugate at dilution of 1: 6000 was applied to the membrane, incubated for 1.5 h at room temperature with gentle agitation. This was followed by washing and the membranes were washed again and the proteins were subsequently visualized with a chemiluminescence kit (ECL) on X-ray film.

### Ouchterlony radial immunodiffusion

To perform this method agar media was prepared by melting 2 g of agar in 50 mL buffer solution (50 mM PO_4_^−^, 75 mM NaCl at pH 7.5) and diluting with 50 mL water. 450 μL of IgG were added to this solution. Petri dishes were used as plates for the test. Several holes were made in the solid agar solution to inject fractions and standards for comparison. Commercial protein A (Repligen, USA) (0.5 mg/mL) was used as a standard.

### Mass spectrometry

The purity and characterization of AviPure eluted from SEC was analyzed by mass spectrometry (Bruker Daltonik GmbH) (MALDI-TOF). 2, 5-dihydroxybenzoic acid (DHB) was applied as matrix solution and the Bruker PepMix Calibration Standard (# 222570, Bruker Daltonics) for the calibration of the instrument. For preparation of the matrix on the AnchorChip the DHB matrix layer method was used (for details see also AnchorChip Manual and Bruker Daltonics product sheets). The Autoflex II software was implemented for the evaluation of samples.

## Results

### Protein engineering

To design the AviPure ligand, the gene fragment coding for the B domains was successfully PCR amplified using the primers producing a PCR gene product of about 407 bp, restricted digested and was in frame ligated into pET28a (+) vector to obtain the expression vector pAV01. In the final vector plasmid pAV01, the B domain sequences were arranged in series, and later transformed into *E.coli* BL21 (DE3) to obtain the engineered *E. coli* BL21 (DE3):pAV01 that was further used in soluble protein expression.

### Mini-pilot scale fermentation of protein A AviPure

Batch fermentations in baffled flasks (Nalgene) were performed to examine the effects of medium composition on cell growth and biomass production in recombinant *E. coli*:pAV01. Initially, high density biomass and protein level expression was compared in five different 1 L shaken culture media to help determine the most favorable media and optimal growth conditions for higher quantity protein and biomass production. During shaking cultivation, increase in cell density was observed in complex media type TB (Table 1), when using the baffled Erlenmeyer flask type. The total protein concentration from the collected 1 mL cell pellets obtained from the overall cultures was determined using the bicinchoninic acid (BCA) assay from Pierce (Rockford, USA) and protein expression was further analyzed using 15 % SDS-PAGE. From Table 1, the results show high total protein and biomass production in LB and TB, with TB producing even higher, while less biomass production was observed in mineral salt media MMM, M9 and RMM. Higher protein content (per gram of cell mass) was observed in complex media containing rich nutrients like yeast extracts than in synthetic media. However, the total protein/biomass ratio for all the media was nearly the same.

**Table 1:** Comparison of different media on total protein and biomass production by *E. coli* BL21 (DE3)/pAV01

Media	Total proteins (g/L)	Biomass (g/L)	Total protein/biomass ratio
LB	2.10 ± 0.12	10.22 ± 0.27	0.21
Terrific broth	3.02 ± 0.25	14.60 ± 0.95	0.21
M9 minimal medium	1.40 ± 0.11	7.45 ± 0.50	0.19
Modified minimal medium	1.63 ± 0.11	8.25 ± 0.38	0.20
Riesenberg mineral medium	1.57 ± 0.07	7.58 ± 0.33	0.21

**Fig. 1:**
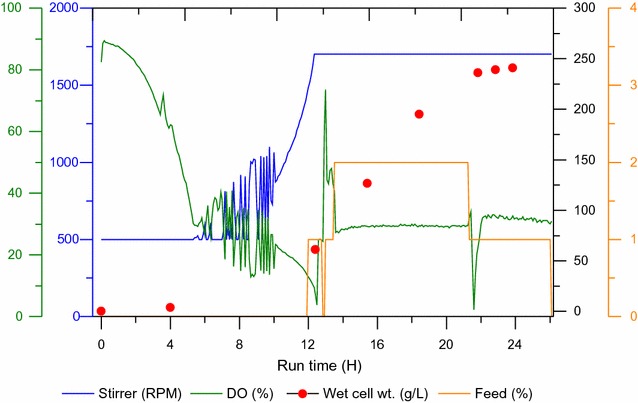
Optical density (OD) and biomass change in time during fermentation. X-axis shows time in hours. Y-axis at the left side shows stirrer speed (RPM) and dissolved oxygen concentration (%) and Y-axis at the right side shows biomass accumulation and feed over the time. Accretion of biomass is related with increase of OD_600_

Based on the shaking culture results, TB, a complex medium and MMM, a synthetic medium (Table 1) were used in mini-pilot scale fermentation. During fermentation, batch culture, then followed by fed batch techniques were used to obtain high cell densities. Biomass production was monitored during cultivation at OD_600_, and from Fig. 1 it can be observed that the feed rate increased exponentially to maintain a specific growth rate at 37 °C. Feed media (glucose) was added to introduce a carbon source and for the purpose of achieving higher cell density. The feed rate adjustments were done according to observations in the previous shake flask studies. After 22 h of cultivation, feed was reduced and culture was induced with 1 mM IPTG final concentration for the induction of the T7 promoter-mediated gene expression and for the production of AviPure. From Fig. 1, the relationship between growth rates in time and change in biomass can easily be noticed as the exponential growth phase between 12 and 22 h is the most remarkable. During fermentation, it was also observed that as the bacterial culture grows pO_2_ saturation decreases; this was due to the increase in O_2_ uptake by the culture. To maintain higher pO_2_ concentration, the stirrer speed was maintained in cascade mode, with the continuously aeration using filter air and by adding feed. Any change in pO_2_ level combined with a pH change triggers the pO_2_ controller. During fermentation, a total of 4800 g of glucose was used, representing 5.6 L injected into the fermenter. The final volume after fermentation was about 15 L. After 25 h of fermentation, product yield of biomass from the media was about 1200 and 2410 g (wet weight) for TB and MMM, respectively. The dry weight obtained from MMM fermentation was about 52 g/L.

**Fig. 2:**
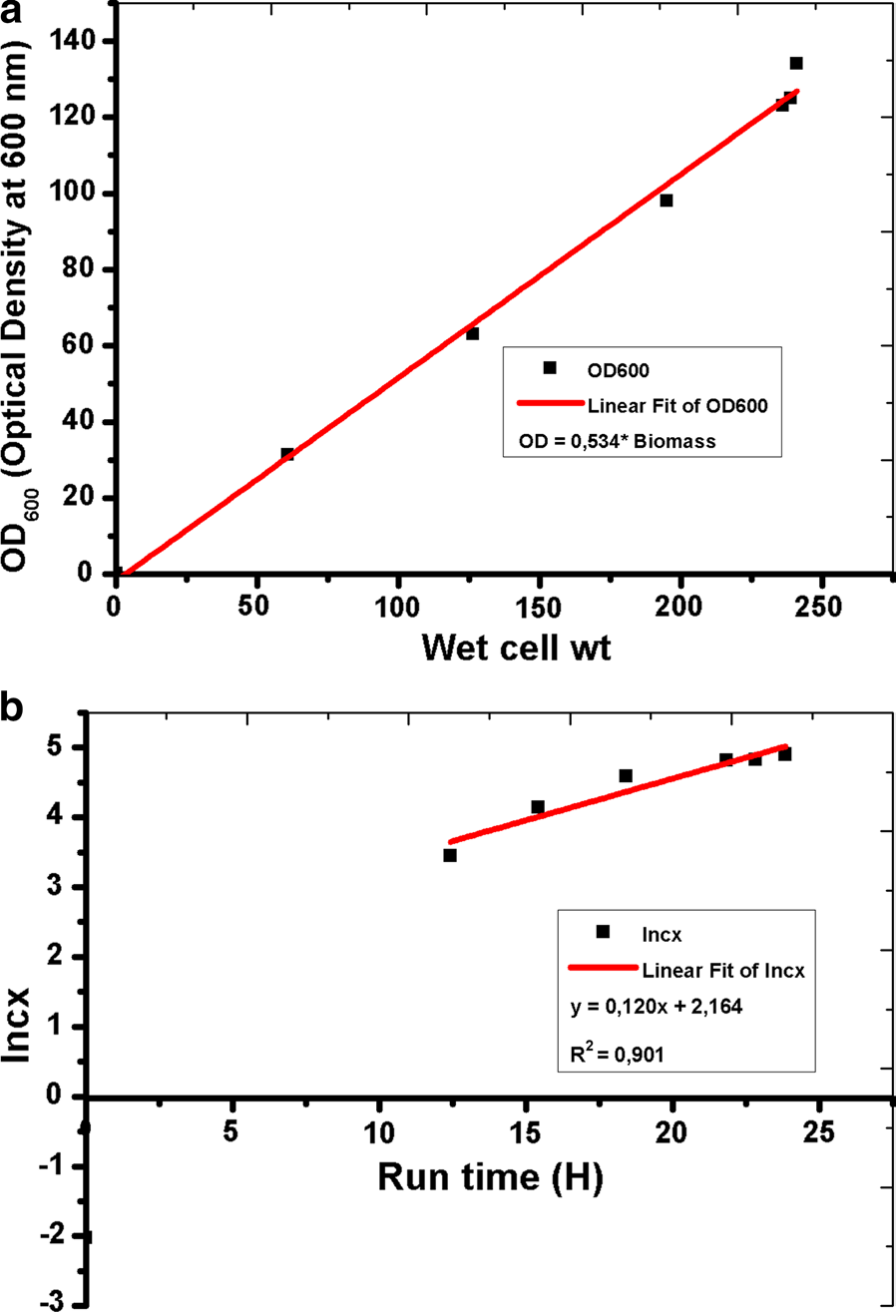
**a** Relationship of OD_600_ to biomass production in *E. coli*. Linear increase of growth rate is shown in the figure by relating biomass and OD_600_. **b** Maximum growth rate calculated by plotting Inc_x_ against time

During fermentation process they are some characteristic phases which affect the procedure. Therefore, to improve the efficiency and yield in fermentation, manipulation of each phase has an effect on the growth of *E. coli* were observed. In fermentation, change in optical density is related to the growth rate of bacterial cells and the results in Fig. 1, clearly shows the relation between *E. coli* growth rate and change of biomass in which the exponential growth phase is the most remarkable and it shows that the inoculation of the culture medium with resultant growth rate at a specific rate (*μ*) increasing from near zero to maximum (*μ*_max_). The results show that the exponential phase is characterized by the exponential growth rate at *μ* = *μ*_max_ and from the extrapolated results (Fig. 2a, b) shows that the specific growth rate for the exponential phase in our study was around 0.3. Additionally, the cell concentration (*c*_x_ in g/L) during fermentation at a given point was calculated by using OD = 0.54 × biomass, equalize to OD/c_x_ = 0.54, previously obtained from the trendline in Fig. 2a. In$(c_{x}) = \mu_{\hbox{max} } \times t - a$ was further extracted from the ln(cx) against the time plot. By using this information, we were able to calculate the starting cell concentration $c_{x,0} = e^{ - a}$ (g/L). Maximum growth rate during the exponential phase was found to be *μ*_max_ = 0.45 (Fig. 2b) and the initial cell concentration is 0.31 (g/L) during fermentation.

### Protein purification and SDS-PAGE gel

**Fig. 3:**
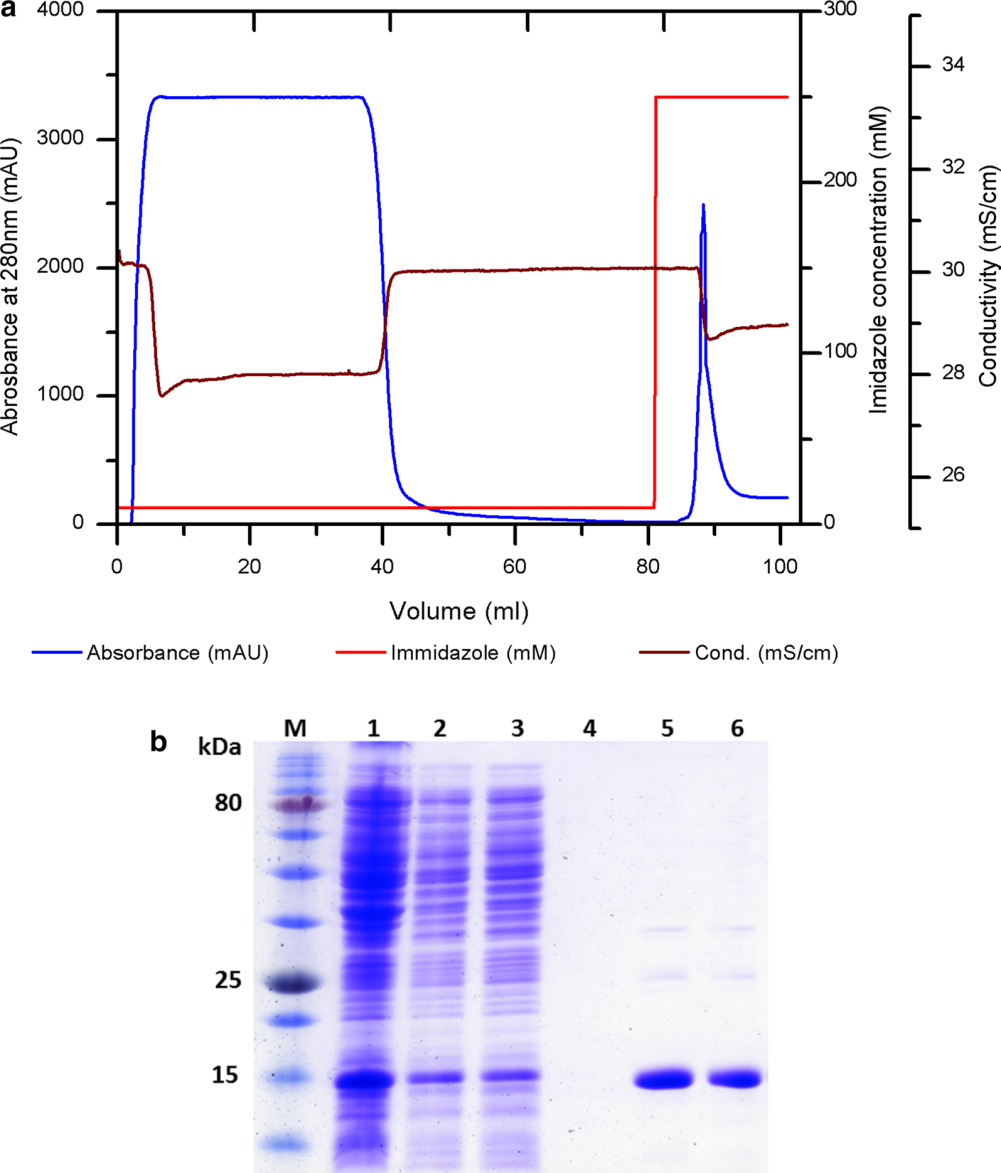
**a** A chromatographic runs of AviPure purification with 5 mL Ni–NTA column, (1) load, (2) wash, (3) elution fraction. **b** *Coomassie Blue* stained 15 % SDS-PAGE gel from different sample fractions, *Lane M* represent pre-stained protein marker, *Lane 1* induced pellets from culture, *Lane 2* supernatant after sonication and centrifugation, *Lane 3* flow through, *Lane 4* Wash, *Lane 5* eluted sample before dialysis, *Lane 6* eluted sample after dialysis

Before chromatographic separation, the sonically lysed cells were centrifuged to remove the insoluble particles and the 40 mL of supernatant was loaded on the Ni–NTA column and the performance of the complete purification process can be seen in Fig. 3a. From the figure, three distinctive stages can clearly be identified. The AviPure elution fractions were pooled, collected and dialyzed. The concentrations of purified protein samples were determined using a Nanodrop 2000c from Thermo Scientific (Wilmington, DE, USA) with the micro volume pedestal and measurement at a wave-length of 280 nm. A total of 1.65 mg/g of protein per gram cell biomass was obtained and the product had a purity of over 90 % as was further confirmed by 15 % SDS-PAGE with coomassie blue staining (Fig. 3b). The process yield of more than 90 % was achieved, though some bands (impurities) of higher molecular weight than AviPure were visible. Due to this, a second purification step using size exclusion chromatography (SEC) (data not shown) was carried out with the aim of getting rid of the impurities.

### Ouchterlony radial immunodiffusion

**Fig. 4:**
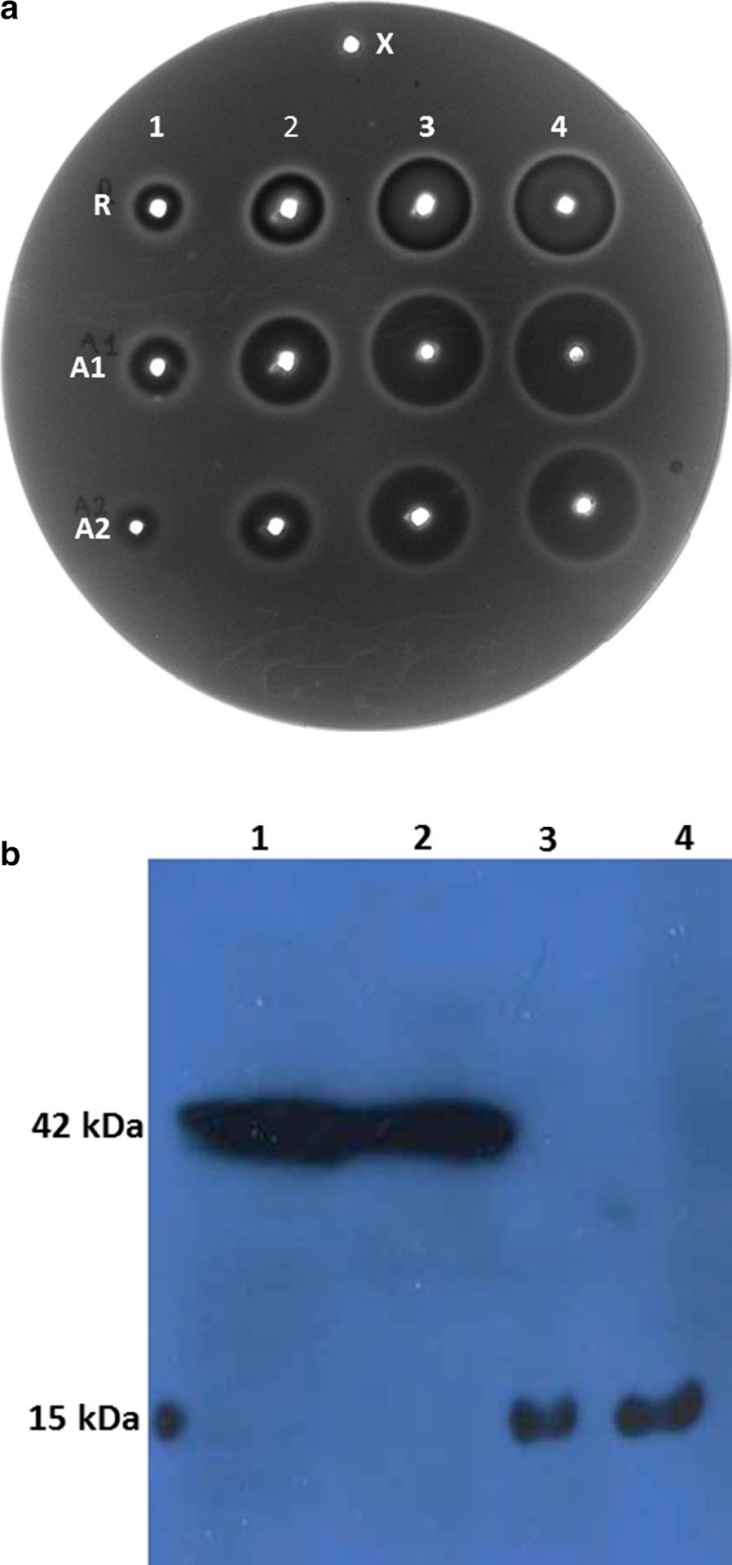
**a** Ouchterlony radial immunodiffusion showing cross-reactivity of IgG with commercial SpA and purified AviPure. *Upper Center* well (*X*) contain BSA. *Upper row* wells (R1–R5) contain different concentration of commercial SpA (concentration 5, 1, 0.5 and 0.1 mg/mL), while middle (A1:1–4) and lower rows (A2:1–4) contain concentration (5, 1, 0.5 and 0.1 mg/mL) of purified AviPure. **b** Western blot analysis of of protein As. Western blot was carried out with Goat anti-protein A polyclonal antibodies HPR conjugate. *Lanes 1 and 2*; Commercial SpA, *Lanes 3 and 4* AviPure eluted from SEC

Ouchterlony radial immunodiffusion analysis assay (Fig. 4a) was performed to check AviPure (A1 and A2) binding activity with immunoglobulin (Octagam, Australia), while commercial protein A designated as R (Repligen, Waltham, MA, USA) was applied as control standard of protein concentration ranging from 5 to 0.1 mg/mL and each analysis was numbered from 1 to 4.

After 24 h of incubation, faint white precipitation rings could be seen on the gels with the naked eye. To amplify these rings, the gel was then stained with Coommassie Blue and the significant dark ring of precipitation is clearly observed, except in well X where BSA was applied.

### IgG affinity detection of AviPure via Western blot analysis

The validity of the purified AviPure was also confirmed by western blot using goat anti-protein A polyclonal antibodies HRP conjugate. The western blot yielded a major band that corresponded to the right molecular weight of the AviPure (Fig. 4b, lanes 3 and 4). No other additional bands were detected by the method, indicating that a pure protein was obtained and no proteolysis occurred during purification and storage.

### Mass spectrometry

**Fig. 5:**
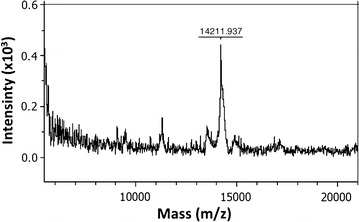
Identification of AviPure expressed in *E. coli* by MALDI-TOF. Purified AviPure from size-exclusion chromatography was used for the analyses

The purified AviPure from SEC showed a single peak in the elution profile fraction, showing that the purity of protein was almost 100 % (Data not shown). In order to investigate SEC results, further analysis of the purified AviPure were conducted by MS MALDI-TOF (Fig. 5) which gave one prominent peak, presenting the AviPure of 14.2 kDa, slightly lower than the mass predicted from the DNA sequence of AviPure (15 kDa), but within the error limit of the technique. This peak proves the molecular weight of the purified AviPure, while peaks below 10,000 m/z (mass/charge) represent background noise from the instrument.

## Discussion

*Staphylococcus aureus* protein A is a highly stable cell surface receptor consists of five domains that have high affinity for the Fc region of IgG and has widely been used mainly in monoclonal antibody purification. In this study, to design the AviPure ligand, we focused on the B domain of SpA, the region having a higher affinity for IgG Fc region (Watanabe et al. 2013) and we were able to demonstrates that the protein can be prepared in high density fed batch fermentation.

During media testing in baffled flasks, it was found that complex media (LB and TB) produced high biomass. In fermentation, media composition is one of the most important parameter that must carefully be considered as cell growth and recombinant product yield depend on it (Shiloach and Fass 2005; Shpigel et al. 2000; Zhang et al. 2004). The cultivation media composition ranges in three major groups: complex, semi complex, and defined. A complex medium contains nutrients which are not well defined chemically e.g., yeast extract, tryptone, peptone, casamino acids, while defined medium consists of chemicals which are chemically defined. Semi defined media is a combination of both complex and defined media nutrients (Panda 2003; Panula-Perala et al. 2008; Schiraldi et al. 2001).

The reason behind high biomass production observed in our batch study when using TB in baffled flasks could have been due to the presence of easy digested yeast extract and tryptone which contains wider varieties of peptides and trace elements, and also due to the glycerol in TB containing a rich carbon source (Schmidt et al. 2005; Eriksen 2008; Sun et al. 2007). On the other hand, fermentation parameters such as specific growth rate could be easily controlled to get a high cell density in the synthetic (mineral) media if carbon rich sources are utilized as fed batch, as can be seen in our results. When mineral salt media is prepared and appropriately supplemented with necessary ingredients, added in the right order and in exact amounts after special sterilization treatments a higher biomass yield production is possible (Freiherr von Roman et al. 2014; Korz et al. 1995; Krause et al. 2010).

During fermentation the pH–stat feeding strategy was used to deal with the issue of glycerol and glucose oscillations based on direct coupling of carbon source consumption and also for the maintenance of related export and import of proton and ammonium ion by the cell during growth. While pH drops by export of H^+^ and import of NH_4_^+^ from the cells to the media by consuming a carbon source, pH increases by import of H^+^ and export of NH_4_^+^ from the cells when the carbon source is exhausted. Although glucose has been used for pH–stat feeding in many fermentation processes, in this study, glycerol and glucose were preferred for high density culture of *E. coli* by pH–stat (Shimizu 2013; García-Arrazola et al. 2005).

Medium composition and growth strategies play the most important role in optimizing high density-cell fermentation processes, as product quality insurance and yield of product are the key issues for large scale fermentations. Process optimization and scale up are aimed to maintain optimum and homogeneous reaction conditions and consequently to minimize stress exposure and enhance metabolic accuracy.

Furthermore, we focused on protein purification and characterization. From the results we can clearly see that the protein was expressed, purified and via western blot, Ouchterlony immunodiffusion assay, the protein maintained its affinity for immunoglobulins.

In this study, high level fed batch fermentation was achieved when using MMM. Several fermentation parameters such as culture growth media, growth conditions and chemical concentrations, pO_2_ level, stirrer speed, pH level and feed media addition were optimized for high density biomass production. The conditions made it possible to culture cells up to 172 g/L wet biomass, (52 g/L dry mass) in 25 h, though Nakano et al. were able to reach cell concentrations of up to 190 and 180 g/L dry cell weight after 30 h fermentation in glucose and glycerol medium (Nakano et al. 1997), respectively. From the reference, the result shows that it possible to obtain higher biomass if continuous feed of carbon source and oxygen available. The final yield of purification process was 1.6 milligrams of AviPure per gram biomass a remarkable result when compared to current literature was achieved. Activity and molecular structures were checked with suitable methods as described before. Results of the characterization experiment showed that after purification steps only a single monomer with a size of 14.2 kDa was present.

The next step is to further characterize and determine the potential application of AviPure for the capture of Immunoglobulin.

## Additional file

10.1186/s13568-015-0155-y DNA nucleotide sequence for Avipure (underlined). Start codons is indicated in bold. Stop codon is highlighted in red.
