# Supplementary material for: High-level fed-batch fermentative expression of an engineered Staphylococcal protein A based ligand in E. coli: purification and characterization
Source: AMB Express. 2015 Nov 10;5:70. doi: 10.1186/s13568-015-0155-y (PMC4641145; doi:10.1186/s13568-015-0155-y)
Supplement: Supplementary file 1 — 10.1186/s13568-015-0155-y DNA nucleotide sequence for Avipure (underlined). Start codons is indicated in bold. Stop codon is highlighted in red. [file 13568_2015_155_MOESM1_ESM.docx]

**AMB Express**

**Additional file**

**High-level fed-batch fermentative expression of an engineered *Staphylococcal* Protein A based ligand in *E. coli*: Purification and characterization**

Martin Kangwa^1^, Vikas Yelemane^1^, Ayse Nur Polat^1^, Kanaka Durga Devi Gorrepati^1^, Mariano Grasselli^2^, and Marcelo Fernández-Lahore^1,^*

^1^*Downstream Bioprocessing Laboratory, Department of Life Sciences & Chemistry, Jacobs University, Campus Ring 1, D-28759 Bremen, Germany.*

^2^*Laboratorio de Materiales Biotecnológicos (LaMaBio), Universidad Nacional de Quilmes-IMBICE (CONICET), Roque Sáenz Peña 352, B1876BXD, Bernal, Argentina.*

^*^Corresponding author: Prof. Dr. Marcelo Fernández-Lahore, Downstream BioProcessing Laboratory, Department of Life Sciences & Chemistry, Jacobs University, Campus Ring 1, D-28759 Bremen, Germany. Phone: +49 421 200 3239, Fax: +49 421 200 3600. E-mail: [m.fernandez-lahore@jacobs-university.de](mailto:m.fernandez-lahore@jacobs-university.de)

**Supplementary Figures 1 (S1)**

| **ATG**CTGGCGGATAATAAATTTAACAAAGAACAGCAGAATGCGTTCTATGAAATCCTGCATCTGCCGAATCTGAACGAAGAACAGCGCAACGCGTTCATTCAGTCTCTGAAAGATGATCCGAGTCAGAGCGCCAATCTGCTGGCCGAAGCGAAAAAACTGAACGATGCGCAGGCCCCGAAAGTGGATAACAAATTTAATAAAGAACAGCAGAACGCCTTTTATGAAATTCTGCATCTGCCGAACCTGAATGAAGAACAGCGTAATGCCTTTATTCAGAGCCTGAAAGATGATCCGTCTCAGTCTGCAAACCTGCTGGCAGAAGCGAAAAAACTGAATGATGCGCAGGCGCCGAAAGGCGGCCATTGCCATTGCCACTAA |
| --- |

**Figure S1** DNA nucleotide sequence for Avipure (underlined). Start codons is indicated in bold. Stop codon is highlighted in red.
